# Data-driven digital health technologies in the remote clinical care of diabetic foot ulcers: a scoping review

**DOI:** 10.3389/fcdhc.2023.1212182

**Published:** 2023-09-01

**Authors:** Joel Lazarus, Iulia Cioroianu, Beate Ehrhardt, David Gurevich, Lisa Kreusser, Benjamin Metcalfe, Prasad Nishtala, Ezio Preatoni, Tamsin H. Sharp

**Affiliations:** ^1^Department of Social and Policy Studies, University of Bath, Bath, United Kingdom; ^2^Department of Politics, Languages and International Studies, Faculty of Humanities and Social Sciences, University of Bath, Bath, United Kingdom; ^3^Institute for Mathematical Innovation, Languages and International Studies, Faculty of Humanities and Social Sciences, University of Bath, Bath, United Kingdom; ^4^Department of Life Sciences, University of Bath, Bath, United Kingdom; ^5^Department of Mathematical Sciences, Faculty of Science, University of Bath, Bath, United Kingdom; ^6^Department of Electronic and Electrical Engineering, Faculty of Science, University of Bath, Bath, United Kingdom; ^7^Department of Life Sciences, Faculty of Engineering and Design, University of Bath, Bath, United Kingdom; ^8^Department for Health, Faculty of Humanities and Social Sciences, University of Bath, Bath, United Kingdom; ^9^Department of Psychology, Faculty of Humanities and Social Sciences, University of Bath, Bath, United Kingdom

**Keywords:** digital health, data science, machine learning, wearable sensors, remote monitoring, telemedicine, diabetes, diabetic foot ulcers

## Abstract

**Background:**

The availability and effectiveness of Digital Health Technologies (DHTs) to support clinicians, empower patients, and generate economic savings for national healthcare systems are growing rapidly. Of particular promise is the capacity of DHTs to autonomously facilitate remote monitoring and treatment. Diabetic Foot Ulcers (DFUs) are characterised by high rates of infection, amputation, mortality, and healthcare costs. With clinical outcomes contingent on activities that can be readily monitored, DFUs present a promising focus for the application of remote DHTs.

**Objective:**

This scoping review has been conducted as a first step toward ascertaining fthe data-related challenges and opportunities for the development of more comprehensive, integrated, and individualised sense/act DHTs. We review the latest developments in the application of DHTs to the remote care of DFUs. We cover the types of DHTs in development and their features, technological readiness, and scope of clinical testing.

**Eligibility criteria:**

Only peer-reviewed original experimental and observational studies, case series and qualitative studies were included in literature searches. All reviews and manuscripts presenting pre-trial prototype technologies were excluded.

**Methods:**

An initial search of three databases (Web of Science, MEDLINE, and Scopus) generated 1,925 English-language papers for screening. 388 papers were assessed as eligible for full-text screening by the review team. 81 manuscripts were found to meet the eligibility criteria.

**Results:**

Only 19% of studies incorporated multiple DHTs. We categorised 56% of studies as ‘Treatment-Manual’, i.e. studies involving technologies aimed at treatment requiring manual data generation, and 26% as ‘Prevention-Autonomous’, i.e. studies of technologies generating data autonomously through wearable sensors aimed at ulcer prevention through patient behavioural change. Only 10% of studies involved more ambitious ‘Treatment-Autonomous’ interventions. We found that studies generally reported high levels of patient adherence and satisfaction.

**Conclusions:**

Our findings point to a major potential role for DHTs in remote personalised medical management of DFUs. However, larger studies are required to assess their impact. Here, we see opportunities for developing much larger, more comprehensive, and integrated monitoring and decision support systems with the potential to address the disease in a more complete context by capturing and integrating data from multiple sources from subjective and objective measurements.

## Introduction

### Rationale

In recent years, the move towards personalised healthcare has accelerated. Integral to this success is the speed of development and the scope of application of Digital Health Technologies (DHTs) (1, 2). Through the integration of sensors, subjective data, software, and computing platforms, DHTs facilitate the development and application of non-clinic-based, remotely located, autonomous, and personalised monitoring, diagnostic, and even therapeutic interventions aimed not just at cure, but, crucially, prevention and overall quality of life improvement (3–6).

One condition for the clinical application of remote DHTs is Diabetic Foot Ulcers (DFUs) (7). DFUs are one of the most common poorly controlled complications of patients with diabetes mellitus and, with a multifactorial aetiology, DFUs require frequent, disruptive, and costly interprofessional interventions (8–12). The academic literature on DFUs reports high rates of: annual incidence (up to 26 million people a year worldwide - 13), mortality (2.5 times higher risk than diabetes alone - 14), infection (over half of all DFUs become infected - 15), and amputation (DFUs precede lower limb amputations in 85% of cases - 16).

DFUs constitute a condition for the application of remote DHTs because four activities - sleep, diet, exercise, and offloading - all play significant parts in prevention, treatment and management of DFUs, and importantly, can be readily remotely monitored and inexpensively altered by the patient without pharmaceutical intervention (17–19). Indeed, recent meta-analyses and review papers note the promise of applying DHTs to prevent DFUs (7, 20, 21). However, they also identify limits to current technological effectiveness in the facilitation of daily monitoring of DFUs and the empowerment of patients and caregivers. Motivated by a belief that an optimal approach to prevention and treatment would be characterised by the application of multiple DHTs designed to be deployed remotely and autonomously and integrated within one accessible interactive application, we conducted this scoping review with a particular focus on studies exploring the integration of multiple technologies and wearable technologies generating autonomous data.

### Objectives

Our aim was to determine the current state of development in the application of DHTs to the remote clinical care of DFUs. We see this as a first step toward subsequently determining the data-related challenges to and opportunities for the design and implementation of more comprehensive, heterogeneous, and potentially individualised sense/act systems, i.e. personalised interventions that adapt to the individual.

We seek to answer two specific questions:

Question 1: What types of technologies populate the field of DHTs used in the remote management of DFUs?

Question 2: What are the issues related to patient experience of DHTs in the remote clinical care of DFUs?

We focus on reported levels of patient satisfaction as a measure for empowering patients in their own management of their disease and wellbeing.

In answering these questions, we aimed to:

- identify the types of DHTs being investigated and the frequency of their respective inclusion in eligible studies.
- ascertain the number of studies that presented single DHTs versus those that trialled the integration of multiple DHTs and their main conclusions.
- compare the proportion of studies focusing on prevention to that of studies focusing on treatment of existing DFUs.

One major potential benefit of remote over clinic-based DHTs is the application of wearable sensors and other technologies for generating data autonomously rather than requiring and relying on the manual interventions of patients or their caregivers. Consequently, we were interested to ascertain how many of the studies included autonomous data generation.

## Methods

### Protocol and registration

A preliminary search of MEDLINE, the Cochrane Database of Systematic Reviews and *JBI Evidence Synthesis* was conducted to ensure no systematic or scoping reviews had been published or were underway that addressed our objectives. A search of the UK National Institute for Health Research International prospective register of systematic reviews (PROSPERO) revealed a systematic review of qualitative studies relating to the application of DHTs to DFU care (22). Our scoping review includes quantitative alongside qualitative studies and focuses on remote care. Our protocol was thus subsequently registered on Figshare.

The rubric of this review adheres to the Participant-Concept-Context (PCC) framework for scoping reviews as prescribed by the Preferred Reporting Items for Systematic Reviews and Meta-Analyses extension for Scoping Reviews (PRISMA-ScR) and Joanna Briggs Institute (JBI) (23–25). The included ‘participants’ are clinicians and patients involved in the trial and use of DHTs in the delivery and receipt of remote clinical care for DFUs. The review’s ‘concept’ is the investigation into technology and data-related challenges to and opportunities for the development of more comprehensive, integrated, and individualised uses of DHTs in the remote care of DFUs. The review’s ‘context’ is the remote care of DFUs.

### Eligibility criteria

Only peer-reviewed original research articles were included in literature searches. All reviews were excluded.

### Information sources

We followed the JBI methodology for scoping reviews guidelines (25). Literature screening was conducted between 1st November 2022 and 5th January 2023 according to the following method. First, in early November 2022, an initial broad search of Medical Subject Headings (MeSH) search terms of three online databases - Medline, Web of Science, and Scopus - was undertaken to generate a shortlist of identified key words and terms relevant to the themes of the application of DHTs in the remote clinical care of DFUs. The contents of this shortlist were agreed among three members of the scoping review team (EP, BM, PN) and a University of Bath librarian. The text words contained in the titles and abstracts of relevant articles, and the index terms used to describe the articles, were used to develop a full search strategy for the same databases. The search terms, including all identified keywords and index terms, were adapted for each database’s unique syntax requirements. The search term for the Web of Science database was as follows:

“digital therap*” OR “digital health” OR “health technolog*” OR “therap* technolog*”) OR biosensor* OR wearable OR biomech* OR “motion capture” OR “movement capture” OR “activity capture” OR track* OR monitor* OR “internet of things” OR “IoT” OR telehealth OR telemed* OR “imag* with internet of things” OR domotic* AND diabet* (foot or feet) ulcer

**Figure 1:**
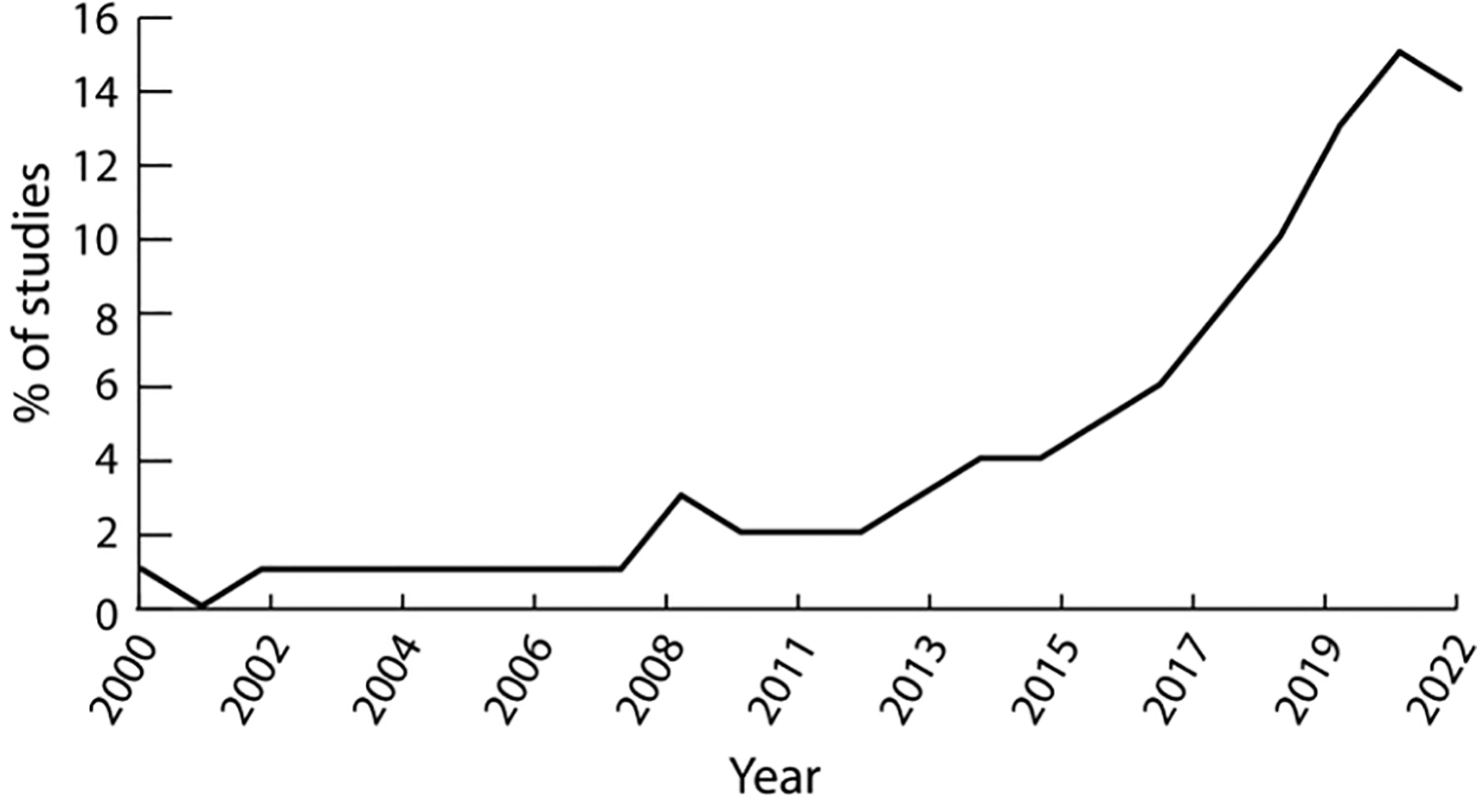
Percentage of publications relating to DHTs and DFUs on Web of Science (2000-2022).

The search was limited to English language literature. A cut-off date of 2010 was imposed on the literature search. This date was deemed to represent a sufficiently long period of time to capture all major DHT developments in the clinical treatment of DFUs. This choice was supported by the rapid rise of publications observed in the area since 2014 (**Figure 1**).

#### Selection of sources of evidence

**Figure 2:**
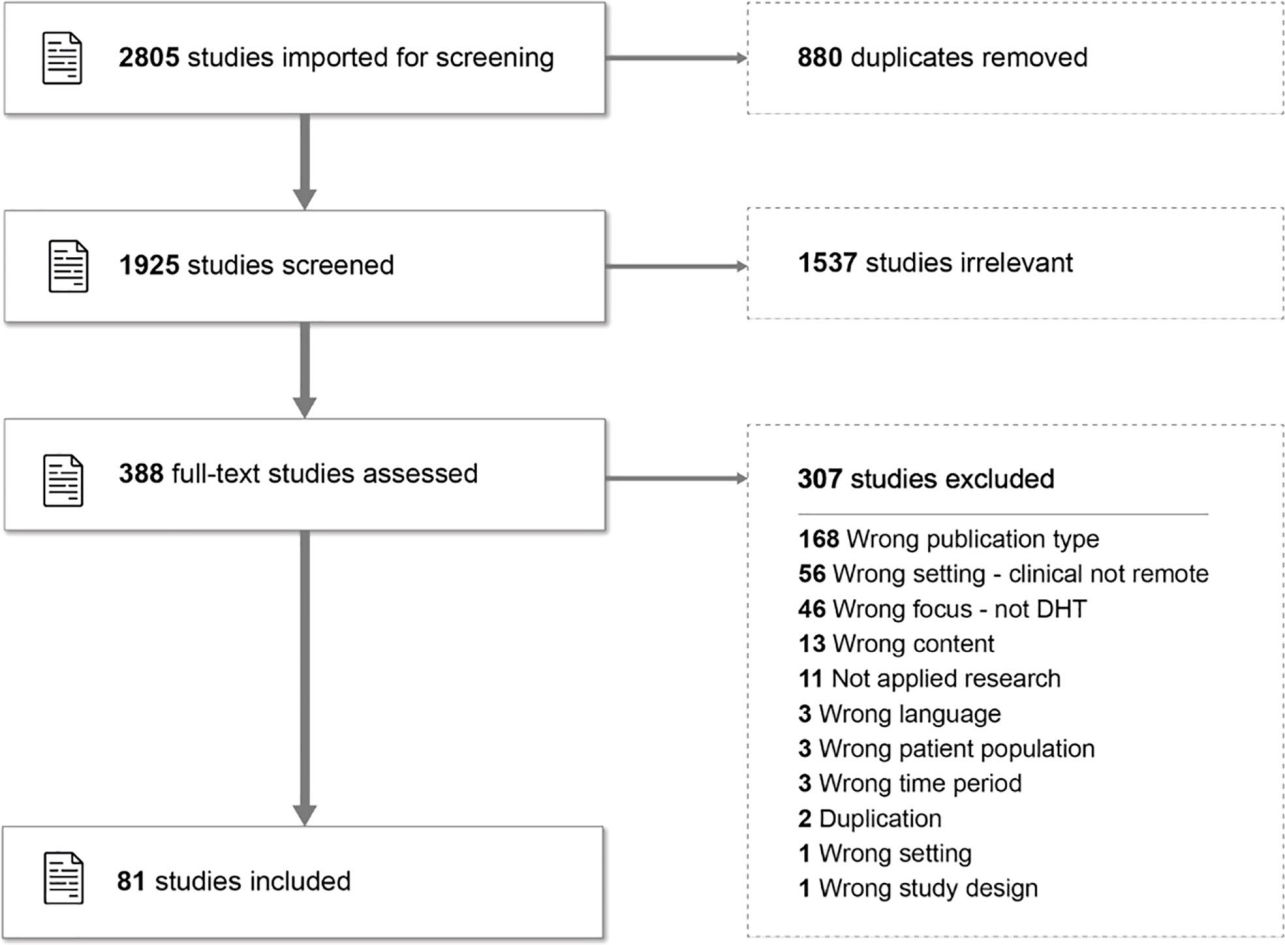
Flow chart of PRISMA-ScR-compliant screening process.

This initial literature search generated 2,805 studies that were then imported into the Covidence platform. Covidence then identified and removed 880 duplicates, leaving 1,925 studies for subsequent screening. JL then conducted the screening process with all remaining team members reviewing over a quarter of the studies’ full texts (randomly selected) to ensure that our eligibility criteria were being effectively applied to the selection. All disagreements were then resolved through discussion between two reviewers and 388 potentially relevant sources were subsequently advanced for full-text review. The full-text review process eliminated 307 papers, leaving 81 papers for data extraction (**Figure 2**, including a summary of exclusion criteria)

### Data charting process and data items

A custom data extraction template was designed in Covidence according to eligibility criteria (**Table 1**).

**Table 1:** Domains for data extraction.

General information	Title
	Lead author contact details
	Country in which study conducted
***Characteristics of study***	Methods
	Aim of study
	Study design
	Study funding sources
	Conflicts of interest?
***Digital Health Technologies***	One or multiple DHT/s?
	Trademarked name of DHT/s?
	Type of DHT
	Description of DHT/s
	Data type, size, format
***Participants***	Total number of participants
	Population description
	Inclusion/exclusion criteria
	Participant demographics
***Study outcomes***	Primary outcome
	Secondary outcome/s
	Conclusions
	Limitations
	Other observations

Once the template was finalised, one reviewer (JL) conducted the data extraction process. The final extracted data were exported, tabled, and summarised to facilitate analysis and identification of trends and themes.

## Results and discussion

### DHT type and frequency

**Figure 3:**
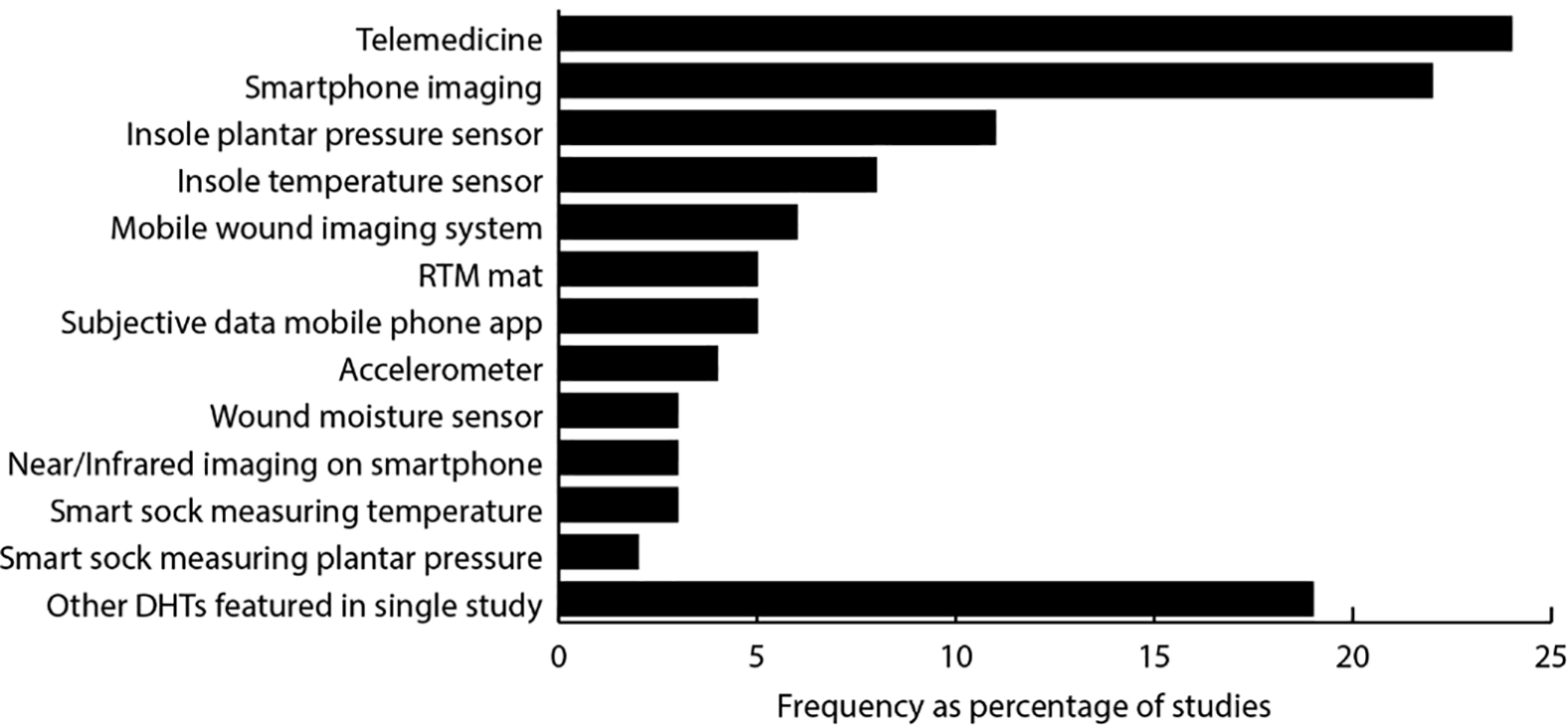
Frequency of appearance of types of DHT in the 81 included studies.

In this section we present a narrative synthesis of the salient study characteristics and findings. We first present the types of DHTs covered and the frequency of their appearance in the 81 studies included (**Figure 3**). Studies of telemedicine^1^ were the most frequent (24% of studies), then smartphone photo imaging (22%), insole pressure sensors (11%), and insole temperature sensors (8%). These four technologies thus comprised the focus of over two-thirds of the DHT studies.

### Single versus multiple DHT studies

The majority (66 out of 81, 81%) of studies referred to a single DHT, whereas only 15 (19%) included multiple DHTs. Of the 66 single DHT studies, 14 were studies of telemedicine, thirteen concerned insole plantar pressure or temperature sensors, and twelve related to smartphone-based imaging. Of the 15 studies that included multiple DHTs, 7 focused on remote treatment and used smartphone/mobile imaging with telemedicine, and 4 focused on warning and prevention and used a combination of wearable sensors (see **Table 2**).

**Table 2:** Categorisations of included studies incorporating multiple DHTs (N=15).

Multiple DHT category	Combination of DHTs	Number of studies	Publications	Objectives
***Remote treatment***	Smartphone/ Mobile imaging with telemedicine	7	(26) (27) (28) (29) (30) (31) (32)	Remote treatment
***Wearables***	Combination of sensors	4	(33) (34) (35) (36)	Warning and prevention

**Table 3:** Characteristics of included studies incorporating multiple DHTs (N=15).

Publication	First author	Types of DHTs combined	Study type	Population size and type
(37)	Foltynski	• Smartphone photo imaging • Glucose meter • Blood pressure monitor	Observational	10 participants (256 images)
(26)	Ladyzynski	• Mobile wound imaging system • Telemedicine	Validation	23 participants (36 wounds)
(27)	Rasmussen	• Smartphone photo imaging • Telemedicine	Experimental	374 participants (DFU patients)
(28)	Smith-Strøm	• Smartphone photo imaging • Telemedicine	Experimental (qualitative RCT)	24 participants (DFU patients)
(38)	Crews	• Accelerometer^2^ • Global Navigation Satellite System	Validation and Observational	10 participants (5 patients at risk and 5 patients with active DFUs)
(33)	Najafi	• Insole pressure sensor • Wound moisture sensor • Insole temperature sensor	Observational	33 participants (Diabetic patients at risk of DFU)
(39)	El-Nahas	• Mat measuring plantar pressure • Smart sock measuring temperature	Validation	25 participants (Healthy volunteers)
(29)	Pak	• Smartphone photo imaging • Telemedicine • Mobile phone app	Observational	60 participants (DFU patients)
(40)	Yavuz	• Temperature- and pressure-regulating insoles	Observational	8 participants (5 healthy; 3 with diabetic neuropathy)
(34)	Du	• Accelerometer • Gyroscope^3^	Proof-of-concept	6 participants (6 diabetic patients recently recovered from DFUs)
(30)	Kong	• Smartphone photo imaging • Telemedicine • Mobile phone app	Observational (case study)	1 participant (DFU patient)
(31)	Main	• Smartphone photo imaging • Telemedicine • Mobile phone app	Proof-of-concept	31 participants (55 ulcers)
(35)	Moulaei	• Insole pressure sensor • Wound moisture sensor • Insole temperature sensor • Mobile phone app	Observational	5 (Diabetic patients without DFUs)
(32)	Cecilia Matilla	• Smartphone photo imaging • Telemedicine • Cloud computing platform	Observational (case study)	1 participant (DFU patient)
(36)	Park	• Accelerometer • Insole temperature sensor • Mobile phone app • Cloud computing platform	Observational	14 participants (Healthy volunteers)

^2^An accelerometer is a motion capture tool that measures the rate of change of the velocity of the object it is attached to. Crews et al. (38) piloted an accelerometer to capture DFU patients’ step data and also to continuously identify body posture (standing, sitting, lying side/prone/supine).

^3^A gyroscope is a device used for measuring or maintaining orientation and angular velocity. Du et al. (34) used a gyroscope to measure gait and balance in elderly DFU patients.

Three other studies share the same goals of the second group of warning and prevention but use a different combination of DHTs. These studies, along with all other multiple DHT studies, are described in greater detail below (see **Table 3**). The only study that trials a wearable technology aimed at moving beyond warning and prevention toward treatment is that of Yavuz et al. (40). Details of this study are provided in a later section of this review.

### A description of the fifteen multiple DHT studies

#### Group 1: remote treatment

Ladyzynski et al. (26) present a mobile foot scanning device called ‘TeleDiaFos’ designed to take and share wound images to enable home-based telemedical care. The authors examined 33 wounds from 23 DFU patients, finding that the images taken and sent by patients with the TeleDiaFoS system were of adequate quality for clinical assessment.

Main et al. (31) piloted another tablet-based application combining wound photo imaging and remote telemedical video conferencing consultations on the NHSNearMe platform with 31 DFU patients. The authors report an 89% rate of healed, improved, or stable ulcers with improved healing in 49.1% (out of 55 ulcers). They estimate potential cost savings of £138,820 and report an elevated level of patient satisfaction. They foresee that ‘an embedded pathway and technology solution including remote consultation can permit early intervention in patients with foot ulcers, with the potential to reduce amputations. However, they recognise the need for expanded studies to validate their preliminary findings.

Rasmussen et al. (27) reported the findings of a randomised controlled trial designed to compare telemedical and standard monitoring of DFU patients among 374 DFU patients in the Region of South Denmark. Results were derived by comparing the number of hospital admissions, inpatient days, ulcer healing, amputations, and deaths. They found no significant difference regarding amputation and healing between telemedical and standard outpatient monitoring. However, the fact that eight of the nine participant deaths occurred in the telemedicine controlled group - a fact that by no means points to explicit causal relationship - leads the authors to call for further studies to investigate effects of telemedicine on all clinical outcomes and patient types.

Pak et al. (29) reported results from an observational study of 60 DFU patients trialling a smartphone-based monitoring and tele-consultation application to establish a standard pressure injury management protocol in a teleconsultation setting to minimise deviation from recommended wound care practices in a non-hospital, teleconsultation setting. Pak et al. (29) found that overall concordance rates between remote and in-person diagnoses were statistically significant for all items and reported elevated levels of satisfaction with the application among patients, caregivers, and clinicians alike.

Cecilia-Matilla et al. (32) presented a case study of a 65-year-old DFU patient) using a smartphone application (‘Healiaco’) to upload wound photo images and subjective data for remote professional monitoring. The system allowed prompt patient referral and the prevention of foot amputation. Greater patient awareness of the importance of treatment adherence was also reported.

Kong et al. (30) presented another case study: the use of the ‘Swift Patient Connect App’ - a similar device to Healico - to effectively treat the ulcer of a 57 year-old type 1 diabetic patient with chronic DFUs. Kong et al. report how, over a one-year period, the application enabled the patient to avoid in-person appointments during the Covid19 pandemic, increased physician confidence in remote wound monitoring, and realised time and cost savings primarily through reduced clinic appointments and nurse visits.

#### Group 2: wearables

Du et al. (34) assessed the feasibility of a ‘wearable sensor-based monitoring system’ to provide an empirical assessment of the relationship between gait and balance changes and the development of foot ulcers in six elderly diabetic patients. They also assess the feasibility of offloading footwear to ameliorate DFU complications. According to the authors, their proof-of-concept study proved ‘convenient, feasible, and effective’ in providing a quantitative assessment of gait and balance changes before and after wearing offloading footwear and thus offers a ‘reliable basis for prognostic management of plantar ulcers’.

Park et al. (36) evaluated a ‘smart offloading boot (SmartBoot) combined with a smartwatch app and cloud dashboard to remotely monitor … adherence and activity’ of 14 healthy adults with active DFUs. This integrated technology combines an accelerometer and insole temperature sensor with the app and cloud computing platform (CCP). The SmartBoot is designed to ‘provide a real-time alert to users when they are out of compliance with their prescribed offloading regimen, prompting improved adherence’. In addition, ‘the clinician dashboard allows the care team to remotely monitor and assess adherence, and provide feedback in near real-time, which could assist in reinforcing adherence to offloading’. Park et al. share data that proves at least the clinical potential of their SmartBoot and report that most trial participants found the SmartBoot ‘easy to use, relatively comfortable, nonintrusive, and innovative’. However, the authors recognise the limited study size of 14 participants, period, and cohort and call for future studies evaluating the ‘clinical validation of real-time non-adherence alerting to improve wound healing outcomes in people with diabetic foot ulcers.

In a proof-of-concept study, Najafi et al. (33) evaluated a ‘smart-textile based on fibre-optics’ woven into a regular sock for ‘simultaneous measurement of plantar temperature, pressure, and joint angles in patients with diabetic peripheral neuropathy ‘. Their ‘SmartSox’ design combines sensors measuring foot temperature, wound moisture, and plantar pressure through an Artificial Neural Network (ANN). By combining three biomedical markers of risk factors ‘inside the hostile environment of the unaltered shoe’ for DFUs, Najafi et al. (33) tested SmartSox on 33 patients with T2DM confirmed diabetic peripheral neuropathy, and a moderate-to-high risk of developing a DFU. The authors report strong results that ‘demonstrates the validity’ of their SmartSox device. They speculate on its potential to ‘provide podiatrists and diabetic foot specialists a unique objective and practical tool to provide a personalised preventive care to manage and prevent diabetic foot at risk of foot ulcers’, but call for a larger sample in a prospective study to validate its clinical application.

In another proof-of-concept study, Moulaei et al. (35) combined plantar pressure, foot temperature, wound moisture sensors within a smart shoe with the aim of monitoring biomarkers to prevent DFUs. The sensors were integrated with an Android-based application that displayed data on patients’ smart phones, provided patients with self-management recommendations, and sent warnings via text to patients when measurements exceeded safe limits and gave recommendations for offloading. The shoe was tested on four diabetic patients at risk of DFU and one healthy adult. Moulaei et al. report accurate performance of the pressure, humidity and temperature sensors, but call for larger studies to validate the system’s efficacy and also to investigate the ‘effectiveness of this system for improving patients’ self-management behaviours and health outcomes’.

#### Other multiple DHT studies

Crews et al. (38) successfully trialled a ‘Physical Activity Monitor’ combining an accelerometer and Global Navigation Satellite System (GNSS) to capture data on ten diabetic participants’ steps, body posture, and standing time to monitor location-specific physical activity. They found that this combination allowed for ongoing and objective logging of location-specific profiles of both those at-risk and those with active DFUs.

Foltynski et al. (37) presented the ‘Patient’s Module’ - a device able to take wound pictures, download data from blood pressure metres and blood glucose meters, and transmit all these data to a central database. The authors evaluated the Patient’s Module on ten patients with DFUs, finding average reduction levels of wound size reduction after twelve weeks. They call for a randomised clinical trial to determine the device’s efficacy.

El-Nahas et al. (39) investigated the proof-of-concept efficacy of their smart sock design in monitoring plantar temperature under real-life conditions to predict plantar pressure distribution. Twenty-five healthy adults participated in wearing the smart socks with recording daily plantar pressure on a plantar pressure recording mat. The authors found that their smart sock design provided autonomous monitoring of plantar temperature changes and a strong correlation between temperature change and plantar pressure distribution. Again, the authors call for larger studies focused on cohorts of patients with or at risk of DFUs.

#### Manual versus autonomous data production

Only 30 of the 81 (37%) studies involved DHTs generating autonomous data from wearable technologies that incorporated sensors. Most studies (63%) were of DHTs, predominantly studies of imaging and telemedicine, that involved the manual generation of data.

‘Treatment-Manual’ versus ‘Prevention-Autonomous’ studies

We found that 55 (69%) were focused on treatment of existing DFU patients while 26 studies (31%) were explicitly focused on prevention. We note the overlap between studies focused on treatment that involved manual data-generation through imaging of ulcers and using telemedicine involving remote online consultations over standard clinic-based consultations. We found that it was predominantly those studies focused on testing remote imaging technologies that measured their efficacy in terms of ulcer image capture accuracy or on reduction in ulcer size.

Twenty-two out of the twenty-six studies focused on prevention involved the application of wearable sensors for autonomous data generation to facilitate monitoring - the other four studies being those evaluating a ‘Remote Temperature Monitoring’ (RTM) mat (41–44). The strategy of prevention in all these prevention-focused studies involves deploying wearable sensors to detect specific dangerous conditions and connected mobile applications to alert patients to stop specific behaviours producing those conditions. It is perhaps obvious, but nonetheless important to note that such a strategy seems indispensable to empowering patients suffering a DFU in optimising their chances for full recovery. This is evidenced by six studies that trial wearable sensors in the service of the active treatment of DFUs (45–50).

**Table 4** captures the frequency of these three categories of study:

**Table 4:** Categorising studies according to treatment/prevention and discrete/continuous binaries.

Type of study	Type/s of DHT	Frequency	Percentage of studies	Total
**Treatment-** **Manual**	Imaging	19	23	46
	Telemedicine	15	18	
	Imaging + Telemedicine	7	9	
	At-home foot skin temperature monitoring	1	1	
	Patient subjective data via mobile phone app	4	5	
**Treatment-** **Autonomous**	Sensors (plantar pressure, temperature, wound moisture)	6	7	9
	Negative pressure wound therapy	1	1	
	Plantar compression and offloading	1	1	
	Wearable pulsed radio frequency electromagnetic energy	1	1	
**Prevention-** **Autonomous**	Sensors (plantar pressure, temperature, wound moisture)	21	26	21
**Prevention-** **Manual**	Remote Temperature Monitoring mat	4	5	5
	Smartphone imaging + glucosometer + blood pressure monitor	1	1	

The Treatment-Manual versus Prevention-Autonomous binary accounts for 67 out of 81 studies (83%) and so captures a central characteristic of current research and development of DHTs for DFUs. Within this statistic, the Treatment-Manual studies focused on photo imaging and telemedicine account for 46 (57%) of this number with 21 (26%) Prevention-Autonomous sensor studies. We can also delineate a trend within Treatment-Manual studies to measure success, unsurprisingly, in relation to image accuracy and/or ulcer size reduction or levels of patient uptake. While studies testing technical efficacy measure success according to the accuracy and reliability of sensor data, more advanced Prevention-Autonomous studies measure success in relation to participant behaviour (changes, adherence).

#### Simple image capture versus image measurement studies

Almost one-third of all studies (n=26) involve the trial of some form of remote imaging technology. The bulk of these studies (18) involve the capture of simple photographic images from a smartphone. Another five studies involve the application of a mobile wound imaging device. Of all 26 image capture studies, only three deploy technologies that are designed not just to capture images, but to measure ulcer size through the use of image processing algorithms. Foltynski et al. (51) tested the accuracy of ulcer measurement of ‘AreaMe’, a ‘new smartphone-based method for wound area measurement’ against two other commercial alternatives, Visitrak and SilhouetteMobile, by using 108 wound shapes that were measured five times with each device, and comparing them to the measurements from an optical scanner. They reported that AreaMe proved more accurate and precise than Visitrak and less accurate and precise than SilhouetteMobile. More recently, Chan et al. (52) sought clinical validation of their ‘CW4’ ‘artificial intelligence-enabled wound imaging mobile application’. They reported ‘excellent’ levels of inter- and intra-rater reliability of their CW4 smartphone image capture and measurement application. Cassidy et al. (53) present and test the usability and reliability of their ‘cloud-based deep learning framework for remote detection’ of DFUs. They report an agreement of 87.69% (i.e., 178 out of 203 images) between application and clinician diagnosis of DFU based on a cohort of 81 patients. An accompanying questionnaire for participant clinicians indicated that all respondents reported high levels of satisfaction with using the app. Cassidy et al. (53) emphasise the novelty of their development of a cloud-based application ‘where DFU can be automatically detected and localized by a fully integrated framework of state-of-the-art technologies with an easy-to-use app, producing high confidence scores, where inference is performed in the cloud’. They see the potential for its expansion to serve both self-monitoring patients and clinicians tasked with diagnosis and monitoring.

#### Smart versus supersmart studies

We distinguished studies using wearables into those studies developing DHTs comprised merely of sensors designed to capture biomarker data for monitoring (i.e., reactive or smart) versus those studies pioneering devices designed not just to sense but to regulate physiological conditions (i.e., supersmart).

Three prospective feasibility studies present active technologies for DFU treatment. Lerman et al. (54) present their ‘SNaP Wound Care System’ that uses negative pressure wound therapy to treat DFU patients. Their case series report full wound closure within between four and eight weeks on chronic wounds of four DFU patients.

Armstrong et al. (55) present the ‘OptiPulse Active Therapy’ device - a ‘unique wearable device that provides intermittent plantar compression and offloading in the treatment of non-healing diabetic foot ulcers’. They report that, in ten patients suffering grade 1 DFUs, eight out of ten wounds healed within twelve weeks of OptiPulse treatment. They deem the device ‘an alternative for safe and effective’ treatment for remote treatment for DFU patients. Rawe et al. (56) present a case series testing ‘ActiPatch’, a ‘wearable pulsed radio frequency electromagnetic (PRFE) energy’ device, on four male in patients over 40 years old with ulcers persisting for over three months that had failed to respond to conventional treatment. They found that within six weeks of regular ActiPatch use, two patients’ ulcers had healed completely and two other patients had a 95% and 88% reduction in wound size. Rawe et al. conclude that their pilot study demonstrated that ‘lightweight wearable PRFE devices may be an effective adjunct therapy for recalcitrant wounds promoting healing and reducing pain’.

Yavuz et al. (40) present data from an observational study of ‘Temperature and Pressure Monitoring and Regulating Insoles’ (TAPMARI) tested on five healthy participants and three participants with diabetic neuropathy. They report that TAPMARI was ‘successful in regulating plantar temperatures at or below the target temperature’ for all eight participants, but that high temperatures in mid-foot range among neuropathic participants pointed to the need for design improvements. With regard to plantar pressure regulation, the authors report that TAPMARI achieved a ‘relatively good pressure distribution’ across participants, achieving levels of pressure lower than those recommended for diabetic footwear. Yavuz et al. calculate that TAPMARI prevented a 40% increase in the metabolic rate in the regulated foot and hence regulated the demand for oxygen in the tissue. They conclude that ‘TAPMARI, after rigorous clinical testing, has the potential to provide better preventive care for diabetic patients’.

#### Technology readiness levels & clinical testing

Since in our screening and full text review processes, we excluded studies of technologies not yet at the stage of testing in human participants, all included studies are of a high technological readiness level (TRL ≥ 5). Overall, we found telemedicine to be the DHT at a high level of clinical testing. This is evidenced by 14 (4 experimental, 10 observational) studies assessing the efficacy of telemedicine versus standard clinic-based care.

Beyond telemedicine, we note that the included studies are considerably further away from widespread clinical testing. We note that only 15 studies (19%) involved a cohort of participants greater than 100 and that 33 studies (41%) are observational in nature. Only seven studies (9%) present results from some form of randomised clinical trial (27, 57–62). An additional five studies report findings from non-randomised control trials (63–67).

### Patient experience

Six studies demonstrate the non-inferiority of telemedicine based on positive patient evaluations and/or adherence rates (68–73). A seventh study extracts secondary data from a large randomised control trial to report similar findings based on Patient-reported Outcome Measures (PROMs) (57). Similarly, a number of studies report high levels of efficacy and satisfaction with capturing and uploading images of ulcers (28, 62, 74, 75) and among clinicians (53). Smith-Strøm et al. (28) conducted semi-structured interviews with 24 DFU patients in the context of a large clustered RCT trialling smartphone imaging and telemedicine. The authors reported that effective telemedical care depended on healthcare professionals’ competence, continuity of care, and, above all, accessibility to services.

With regard to wearable technologies, several studies report good or high levels of patient utilisation and adherence and/or satisfaction for smart socks (76, 77), smart footwear (34, 36) and insole temperature and pressure sensors (45, 61, 78, 79). Scholten et al. (76) report that 160 participants wore their smart socks for 22-25 days per month on average with a retention rate of 91.9% after seven months. Reyzelman et al. (77) report average scores of over nine in a ten-point scale for ease and comfort of use. Park et al. (36) report participants agreeing that Najafi et al. (79) reports average scores of over four in a five point-scale for comfort and benefit for their insole sensors. Ehrmann et al. (48) report low levels of patient adherence for an insole temperature sensor with 26 DFU patients wearing their insoles for about four hours a day.

Regarding the use of mobile phone applications to improve patient behaviour, Schneider et al. (80) report treatment acceptability average scores of 4.79 out of 5 with interviewed patients perceiving benefits from the intervention. Kilic et al. (81) conducted a small randomised pilot study of the ‘m-DAKBAS’ mobile foot care application on ten DFU patients. The application enabled patients to upload subjective data relating to nine aspects of DFUs. The authors report a significant increase in knowledge level, behaviour, and self-efficacy scores in both control and experimental groups as well as high patient satisfaction levels.

## Conclusions and next steps

In this scoping review, we sought to answer two specific questions:

Question 1: What types of technologies populate the field of DHTs used in the remote management of DFUs?
Question 2: What are the issues related to patient experience of DHTs in the remote clinical care of DFUs?

We find that telemedicine and smartphone imaging account for almost half of the technologies used in included studies. We find that only 15 of 81 studies incorporated the use of multiple DHTs. We categorise a majority of studies (56%) as ‘treatment-manual’ (mainly imaging and telemedicine) and over a quarter as ‘prevention-autonomous’ (mainly wearables) (26%). Only 10% of studies involved more ambitious ‘treatment-autonomous’ interventions. And only three studies involved the trial of supersmart technologies. Finally, we also find studies generally reporting high levels of patient adherence and satisfaction which are enhanced by the presence of various factors including measures to educate and support patients.

We thus conclude that what the findings from this scoping review point to is that, despite the relatively high number of publications, there are few studies that are trialling combinations of autonomous data-generating technologies aimed at prevention (26% of studies) and even fewer aimed at treatment (10%). No studies attempt to combine more than three DHTs and thus to combine multiple data sources for diagnosis or personalised intervention. Only four of 81 studies combined objective (from sensors and images) and subjective (via mobile phone apps) data (30, 31, 35, 36).

Our scoping review was limited to the types of DHTs being deployed in the field of DFU care and the degree of technological readiness, clinical testing, and patient experience. An investigation into the major issues of clinical efficacy, economic effectiveness, and data analytic methods and challenges lie ahead. The review clearly shows that realising the potential of DHTs in delivering remote, personalised treatment and prevention of DFUs will require larger studies to evaluate their impact. Nonetheless, the promising levels of technological accuracy and reliability, and, in general, of patient adherence and satisfaction that most of such studies report tell us that there is indeed an opportunity for developing much larger, more comprehensive, and integrated monitoring and decision support systems with the potential to address the disease in a more complete context by capturing and integrating data from multiple sources from subjective and objective measurements. Such a system would include wearables, home-consumer devices, and social interaction apps. Such an approach would capitalise on novel deep learning algorithms (e.g., classification of movements within a real-life environment, identification of protective vs adverse behaviours) that can classify an individual’s data in real-time by simultaneously modelling behavioural big data from various sources and formats whilst accounting for both the current snapshot of a patient and their trajectory over time. This information could then be integrated from real-time remote monitoring to decision support systems for clinicians which will enable the generation of real-time interventions tailored to the needs of the individual patient.

We recognise the major challenges associated with the sharing and integration of data from heterogeneous sources that our envisioned approach to DHT would encounter. The UK’s National Health Service (NHS) is an exemplary case study. For example, the UK Parliament Health and Social Care Committee’s (82) recent report on Digital Transformation in the NHS states, “[t]he NHS faces significant challenges in integrating data from various sources, hindering the seamless delivery of digital healthcare services”.

Our review focused on the types of technologies under development rather than on the challenges associated with deployment and integration, and, since fewer than a fifth of studies covered integrated multiple technologies, very few alluded to the integration challenges. We plan to conduct further studies exploring issues such as the ability to capture large amounts of patient data; the ease of transferring data (bandwidth and other related software) between the physician and patient; and the ease of storing, accessing, and sharing the data (i.e. cloud storage). These three issues are fundamental to developing technical platforms capable of delivering remote care.

The fact that Kerr et al. (83) found that 60% of NHS England expenditure on DFUs was for care in community, outpatient and primary settings speaks to the need in the UK for the development of DHTs capable of supporting both remote prevention and treatment. This will require the development of integrated DHTs designed to generate and integrate both manual and autonomous data.

## Data availability statement

The original contributions presented in the study are included in the article/supplementary material. Further inquiries can be directed to the corresponding author.

## Author contributions

JL was the primary researcher and author of the scoping review. Every other author contributed equally to the research design and drafting and editing of the review.
